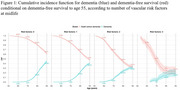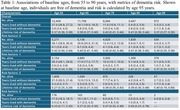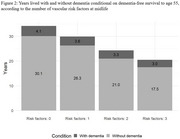# Association of Midlife Vascular Health with Dementia‐Free Life Expectancy and Years Lived with Dementia

**DOI:** 10.1002/alz70860_104468

**Published:** 2025-12-23

**Authors:** Jordan Weiss, Jiaqi Hu, Richey Sharrett, Rebecca F. Gottesman, Pamela L. Lutsey, Thomas H. Mosley, Elizabeth Selvin, Michael Fang, Josef Coresh

**Affiliations:** ^1^ NYU Grossman School of Medicine, New York, NY, USA; ^2^ Tsinghua University, Beijing, Haidian District, China; ^3^ Johns Hopkins University Bloomberg School of Public Health, Baltimore, MD, USA; ^4^ National Institute of Neurological Disorders & Stroke, Bethesda, MD, USA; ^5^ University of Minnesota School of Public Health, Minneapolis, MN, USA; ^6^ University of Mississippi Medical Center, The MIND Center, Jackson, MS, USA; ^7^ Johns Hopkins Bloomberg School of Public Health, Baltimore, MD, USA; ^8^ Departments of Population Health and Medicine, New York University Grossman School of Medicine, New York, NY, USA

## Abstract

**Background:**

Midlife vascular risk factors, including diabetes, hypertension, and smoking, are associated with increased risks of dementia and mortality. While prior research has focused on individual risk factors, the joint burden multiple risk factors on dementia onset and years lived with and without dementia remains unclear. Understanding these relationships is critical for informing interventions to promote healthy aging.

**Method:**

We conducted a prospective cohort analysis of 12,409 participants in the Atherosclerosis Risk in Communities (ARIC) Study, stratified by the number of midlife vascular risk factors (0, 1, 2, or 3). Diabetes was identified by self‐report, medication use, or HbA1c ≥6.5%. Hypertension was defined as blood pressure ≥140/90 mmHg or medication use. Smoking status was self‐reported, with non‐current smokers as the reference. We estimated cumulative incidence functions for dementia and death before dementia and calculated years lived event‐free and with dementia using the Kaplan‐Meier method. Cause‐specific hazard models assessed the association of vascular risk burden with these outcomes.

**Result:**

Among the 12,409 participants (mean age, 56.2 years [SD 5.2]; 56.0% female; 25.2% Black adults), 57.5% had at least one midlife vascular risk factor. At age 55, individuals with no risk factors were expected to live 30.1 dementia‐free years (95% CI, 29.9–30.4), compared with 17.5 years (95% CI, 16.3–18.7) for those with three risk factors. The lifetime risk of dementia was higher among individuals with no risk factors (42.0%; 95% CI, 40.0%–44.0%) than among those with three risk factors (23.0%; 95% CI, 17.0%–30.0%) due to increased mortality with greater vascular burden. Among individuals who developed dementia, those with no vascular risk factors lived a mean of 4.1 years with dementia (95% CI, 3.8–4.4) versus 3.0 years (95% CI, 2.0–4.1) for those with three risk factors.

**Conclusion:**

A higher burden of midlife vascular risk factors is associated with earlier dementia and a reduction in dementia‐free life expectancy by up to 13 years with an additional 2‐4 years with dementia, this is primarily due to higher mortality. These findings highlight the urgent need for midlife vascular health interventions to delay dementia onset and promote brain health.